# Whole‐Genome Characterization of Rare Artiodactyl‐Like G10P [14] and G8P [14] Rotavirus a Strains Detected in Pediatric Gastroenteritis Cases in Hokkaido Prefecture, Japan

**DOI:** 10.1002/jmv.70875

**Published:** 2026-03-18

**Authors:** Akira Takebayashi, Yuya Fukuda, Yoshiki Fujii, Hiroshi Nihira, Minako Kihara, Yoshimasa Kudou, Shuhei Adachi, Noritaka Shintani, Takeshi Tsugawa

**Affiliations:** ^1^ Department of Pediatrics Sapporo Medical University Sapporo Japan; ^2^ Department of Virology II National Institute of Infectious Diseases, Institute for Health Security Tokyo Japan; ^3^ Department of Pediatrics Nihira Children's Clinic Sapporo Japan; ^4^ Department of Pediatrics Tomakomai City Hospital Hokkaido Japan

**Keywords:** artiodactyl rotavirus, gastroenteritis, human rotavirus, interspecies transmission, Japan

## Abstract

*Rotavirus alphagastroenteritidis* (RVA) is the leading cause of acute gastroenteritis in young children worldwide, as well as in a wide range of animal species. In 2020 and 2022, we identified three unusual RVA strains in pediatric gastroenteritis patients in Hokkaido Prefecture, Japan, using next‐generation sequencing: one G10P[14] strain (Ni20‐23) and two G8P[14] strains (Ni20‐37 and To22‐1). All three strains possessed the genotype constellation G10/G8‐P[14]‐I2‐R2‐C2‐M2‐A3‐N2‐T6‐E2‐H3. Phylogenetic analyses of these 11 genome segments indicated that the strains were artiodactyl‐like RVAs. However, no genome segment exhibited > 98% nucleotide identity among the three strains, suggesting that they originated from distinct artiodactyl RVA strains. Furthermore, no previously reported artiodactyl RVA strain showed > 90% nucleotide identity across all 11 segments; thus, the direct animal origins of these strains remain unclear. As observed in these strains, nearly all artiodactyl‐like RVAs previously detected in humans have carried the VP4 genotype P[14], indicating a potential role of P[14] in facilitating cross‐species transmission. One strain (To22‐1/G8P[14]) was detected in a child who had received two doses of Rotarix (G1P[8] monovalent vaccine). Comparative amino acid analysis of VP4 between the To22‐1/G8P[14] strain and the P[8] Rotarix vaccine strain revealed substitutions at 18 of 37 (48.6%) known antigenic epitope sites, suggesting a potential antigenic mismatch. Continued surveillance and further studies are warranted to elucidate the mechanisms underlying interspecies transmission of artiodactyl‐like P[14] RVAs and to assess the effectiveness of current vaccines against such strains.

## 1 Introduction

*Rotavirus alphagastroenteritidis* (RVA) is the leading cause of acute gastroenteritis in young children worldwide, as well as in various animal species. RVAs belong to the family *Sedoreoviridae* and are non‐enveloped, icosahedral viruses with a genome consisting of 11 segments of double‐stranded RNA. These segments encode six structural proteins (VP1 to VP4, VP6, and VP7) and six nonstructural proteins (NSP1 to NSP6) [1].

Current RVA classification, based on the genotyping of all 11 genome segments, shows that most human strains fall into two major genotype constellations: the Wa‐like constellation (G1/3/4/9‐P[8]‐I1‐R1‐C1‐M1‐A1‐N1‐T1‐E1‐H1) and the DS‐1‐like constellation (G2‐P[4]‐I2‐R2‐C2‐M2‐A2‐N2‐T2‐E2‐H2) [2, 3]. Two oral RVA vaccines, Rotarix and RotaTeq, were introduced in 2006, and the global RVA landscape subsequently shifted, with emerging genotypes such as DS‐1‐like G1P [8], equine‐like G3P [8], G8P [8], and G12P [8] increasingly detected [4, 5, 6, 7, 8]. Nevertheless, these novel strains generally retain either the Wa‐like or DS‐1‐like genomic backbone. Human RVA strains with genotype constellations markedly divergent from these two backbones are rare and often associated with zoonotic transmission from animal reservoirs. In the post‐vaccine era, the emergence of animal‐derived RVA strains with divergent genetic and antigenic properties may represent a potential challenge to current control strategies and highlights the importance of integrated human–animal surveillance from a One Health perspective.

We have conducted continuous molecular epidemiological surveillance of RVA in Hokkaido Prefecture, Japan, since the 1980s and have genetically analyzed approximately 2000 stool samples from patients with RVA gastroenteritis up to 2019 [9, 10, 11, 12]. In this surveillance, we identified only a single P [14] strain (Ni17‐46) from a 15‐year‐old girl with diarrhea in 2017 [13]. The P [14] VP4 genotype is occasionally found in artiodactyl species such as cattle, deer, sheep, and goats, but remains rare in humans, suggesting sporadic interspecies transmission events [14].

In Japan, Rotarix and RotaTeq were introduced for voluntary immunization in 2011 and 2012, respectively, and both were incorporated into the national immunization program in October 2020. Widespread vaccine uptake, together with stringent infection control measures introduced in March 2020 during the COVID‐19 pandemic, led to a substantial decline in RVA gastroenteritis cases in Hokkaido Prefecture through 2022 [15]. This reduction in circulation may have facilitated the detection of rare RVA strains that are typically masked by more prevalent human genotypes.

During this period, we identified three unusual RVA strains in pediatric gastroenteritis patients using next‐generation sequencing (NGS): one G10P [14] strain in 2020 and two G8P [14] strains in 2020 and 2022. In this study, we determined the complete genotype constellations of all 11 genome segments of the three P [14] strains and performed phylogenetic analyses to investigate their evolutionary origins. We also compared amino acid residues at VP7 and VP4 antigenic epitopes between these strains and vaccine strains included in Rotarix and RotaTeq to assess antigenic differences.

## 2 Materials and Methods

### 2.1 Sample Information and RNA Extraction

Between 2020 and 2024, stool specimens were collected from 18 pediatric patients diagnosed with rotavirus gastroenteritis by a rapid antigen detection test at clinics and hospitals in Hokkaido Prefecture, Japan. Clinical information, including age, underlying conditions, rotavirus vaccination history, and clinical symptoms, was retrieved from medical records. All stool samples were subjected to NGS analysis. Viral RNA was extracted from 10% (w/v) stool suspensions with the Direct‐zol RNA MiniPrep kit (Zymo Research, Irvine, CA, USA) according to previously reported methods [16, 17].

### 2.2 Next‐Generation Sequencing (NGS)

NGS was performed as previously described [16, 17, 18, 19]. Briefly, a 200‐bp fragment library was constructed for each sample using the NEBNext Ultra RNA Library Prep Kit for Illumina v1.2 (New England Biolabs), according to the manufacturer's instructions. Library purification was performed using Agencourt AMPure XP magnetic beads (Beckman Coulter) according to the manufacturer's instructions. DNA concentrations were determined on a Qubit 2.0 fluorometer using the Qubit HS DNA Assay (Invitrogen). Paired‐end sequencing (2 × 151 bp) was performed on an iSeq. 100 Sequencing System (Illumina) using the iSeq. 100 i1 Reagent v2. Sequence data were analyzed using CLC Genomics Workbench Software v25.0.1 (CLC Bio). The sequences of the RVAs used in this study were submitted to the DNA Data Bank of Japan (DDBJ) under accession numbers LC898664–LC898696.

### 2.3 Genotyping and Phylogenetic Analysis

The genotype of each of the 11 genome segments was determined with the Rotavirus A Genotype Determination online classification tool (https://www.rivm.nl/mpf/typingtool/rotavirusa/). All 11 genomic segments were separately aligned using the MUSCLE algorithm implemented in MEGA 11, and phylogenetic trees were subsequently constructed with the maximum likelihood method based on Kimura 2‐parameter model and 1000 bootstrap replicates in the same software (https://www.megasoftware.net). Representative reference sequences were selected for comparison from GenBank at the National Center for Biotechnology Information (https://www.ncbi.nlm.nih.gov/nucleotide/). Nucleotide sequence identities were calculated using the ClustalW online alignment tool (https://www.genome.jp/tools-bin/clustalw).

Because no universally accepted criteria exist for defining rotavirus lineages, lineages in this study were defined empirically based on clustering patterns in maximum‐likelihood phylogenetic trees and overall nucleotide identity, and are used solely for descriptive purposes in this study.

### 2.4 Ethics Statement

This study was approved by the Ethics Committee of Sapporo Medical University (approval number: 342‐3458). All procedures involving human participants were conducted in accordance with the ethical standards of the institutional and national research committees and complied with the Declaration of Helsinki (as revised in 2013). Written informed consent was obtained from the parents of all patients.

## 3 Results

Among the 18 samples analyzed, two strains were identified as G8P [14] (Ni20‐37 and To22‐1) and one strain as G10P [14] (Ni20‐23). The average sequencing coverage depths across the 11 genome segments ranged from 664 to 1062 for Ni20‐23, 165 to 261 for Ni20‐37, and 846 to 1689 for To22‐1. The remaining samples consisted of one G1P [8] strain, seven G2P [4] strains, two G3P [8] strains, three G8P [8] strains, and two samples that could not be genotyped.

Ni20‐23 (G10P[14]) was obtained from a 13‐year‐old patient, and Ni20‐37 (G8P[14]) was obtained from a 4‐year‐old patient with RVA gastroenteritis at Nihira Children's Clinic in Sapporo City in 2020 (Table 1). To22‐1 (G8P[14]) was identified from a 5‐year‐old patient with RVA gastroenteritis at Tomakomai City Hospital in 2022. Information on prior animal contact was not available for any of these patients. The To22‐1 patient had a documented history of receiving two doses of Rotarix, whereas the other two patients were unvaccinated. The To22‐1 patient required hospitalization due to a relapse of nephrotic syndrome; however, diarrhea and vomiting occurred only once each, and intravenous fluid therapy was not required. No other common gastrointestinal pathogens were detected in any of the patients.

**TABLE 1:** Characteristics of strains Ni20‐23, Ni20‐37, and To22‐1.

Strain	Patient age	Specimen collection date	City	Clinical Settings	Underlying disease	Rotavirus vaccine	Fever^a^	Vomiting	Diarrhea	GP genotype
Ni20‐23	13 y 11 m	Aug 24, 2020	Sapporo	Outpatient	None	Unvaccinated	Peak 38.2°C; duration 2 days	Up to 2 episodes/day; duration 1 day	None	G10P[14]
Ni20‐37	4 y 5 m	Dec 2, 2020	Sapporo	Outpatient	None	Unvaccinated	Peak 38.5°C; duration 1 day	None	1 episode/day; duration 1 day	G8P[14]
To22‐1	5 y 9 m	Aug 18, 2022	Tomakomai	Inpatient	Nephrotic syndrome	Rotarix (2 doses)	Peak 38.7°C; duration 1 day	1 episode/day; duration 1 day	1 episode/day; duration 1 day	G8P[4]

^a^ Fever was defined as a body temperature of at least 37.5°C.

### 3.1 Whole‐Genome Analysis

**FIGURE 1:**
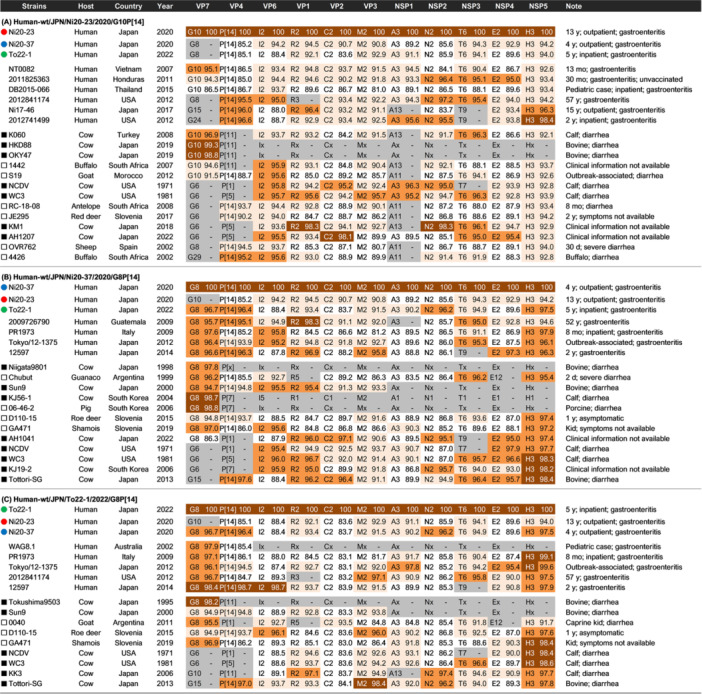
Complete genotype constellations and nucleotide identities (%) of representative human and artiodactyl strains compared with three study strains. Complete genotype constellations and nucleotide sequence identities (%) of the three study strains compared with representative reference strains. Panels (A), (B), and (C) correspond to the nucleotide sequence identities (%) to strains Ni20‐23, Ni20‐37, and To22‐1, respectively. Nucleotide sequence identities of 98.0%–100%, 95.0%–97.9%, 90.0%–94.9%, and < 90.0% are shown in brown, orange, pale orange, and unshaded, respectively. The gray‐shaded cells indicate genotypes that differ from those of the top‐listed study strains (Ni20‐23, Ni20‐37, and To22‐1).

Strain Ni20‐23 had the genotype constellation G10‐P[14]‐I2‐R2‐C2‐M2‐A3‐N2‐T6‐E2‐H3, whereas strains Ni20‐37 and To22‐1 shared the constellation G8‐P[14]‐I2‐R2‐C2‐M2‐A3‐N2‐T6‐E2‐H3 (Figure 1). The only difference among the three strains was the VP7 genotype (G10 vs. G8). Several genome segments (VP4, VP6, VP2, NSP1, NSP2, and NSP4) showed relatively low nucleotide identity among the three strains (83.6%–89.6%), and none of the genome segments exhibited > 98% nucleotide identity among the strains. Basic Local Alignment Search Tool (BLAST) searches indicated that similar genotype constellations are typically found in artiodactyl RVA strains and occasionally in human P [14] strains.

### 3.2 Ni20‐23 (G10P[14])

Ni20‐23 had the same genotype constellation across all 11 genome segments with three previously reported human G10P [14] strains: VNM/NT0082/2007, HON/2011825363/2011, and THA/DB2015‐066/2015 [20, 21], but no genome segment exceeded 98% nucleotide identity with these strains (Figure 1).

### 3.3 Ni20‐37 (G8P[14])

Ni20‐37 had the same genotype constellation across all 11 genome segments as two human G8P [14] strains: JPN/Tokyo/12‐1375/2012 and ITA/PR1973/2004 [22, 23], but no genome segment exceeded 98% nucleotide identity (Figure 1). Ni20‐37 exhibited high nucleotide identity to the Japanese bovine strain JPN/Tottori‐SG/2013, including 98.4% identity in NSP5 and > 95% identity in five other segments (VP4, VP1, VP2, NSP3, and NSP4) [24].

### 3.4 To22‐1 (G8P[14])

To22‐1 also had the same genotype constellation across all 11 genome segments as human G8P [14] strains Tokyo/12‐1375 and PR1973, but no genome segment showed > 98% nucleotide identity (Figure 1) [22, 23]. The VP7, VP4, and VP6 genes showed ≥ 98.4% identity to the human G8P [14] strain JPN/12597/2014 [25].

### 3.5 Phylogenetic Analysis

**FIGURE 2:**
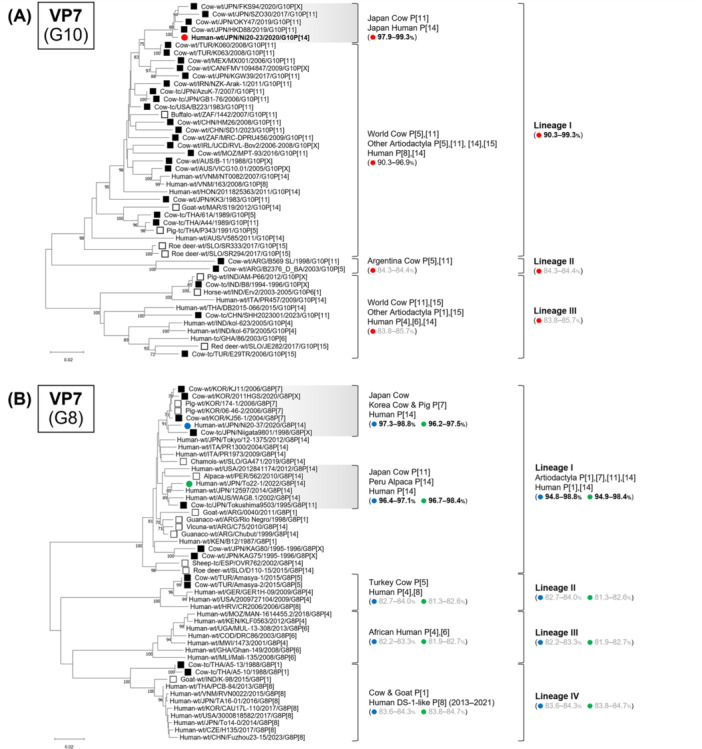
Phylogenetic trees of the VP7 (G10 and G8) genes of the study strains and representative RVA strains. Panel (A) shows the VP7 (G10) gene, and panel (B) shows the VP7 (G8) gene. The phylogenetic trees were constructed with the maximum likelihood method and 1000 bootstrap replicates, using the MEGA 11 software package. Bootstrap values of ≥ 70% are indicated at each node. The genetic distance (nucleotide substitutions per site) is indicated at the bottom of the trees. Strains Ni20‐23, Ni20‐37, and To22‐1 are represented by red, blue, and green filled circles, respectively. Bovine strains and other artiodactyl strains are represented by black‐filled squares and black squares, respectively. Nucleotide identities between strains Ni20‐23, Ni20‐37, and To22‐1 and other strains are shown in parentheses.

**FIGURE 3:**
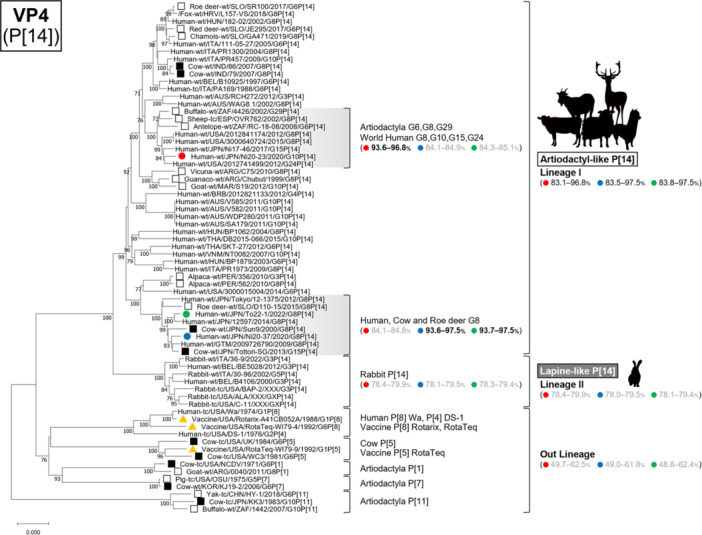
Phylogenetic trees of the VP4 (P[14]) gene of the study strains and representative RVA strains. The phylogenetic trees were constructed with the maximum likelihood method and 1000 bootstrap replicates, using the MEGA 11 software package. Bootstrap values of ≥ 70% are indicated at each node. The genetic distance (nucleotide substitutions per site) is indicated at the bottom of the trees. Strains Ni20‐23, Ni20‐37, and To22‐1 are represented by red, blue, and green filled circles, respectively. Bovine strains and other artiodactyl strains are represented by black filled squares and black squares, respectively. Vaccine strains Rotarix and RotaTeq are represented by orange‐filled triangles. Nucleotide identities between strains Ni20‐23, Ni20‐37, and To22‐1 and other strains are shown in parentheses.

**FIGURE 4:**
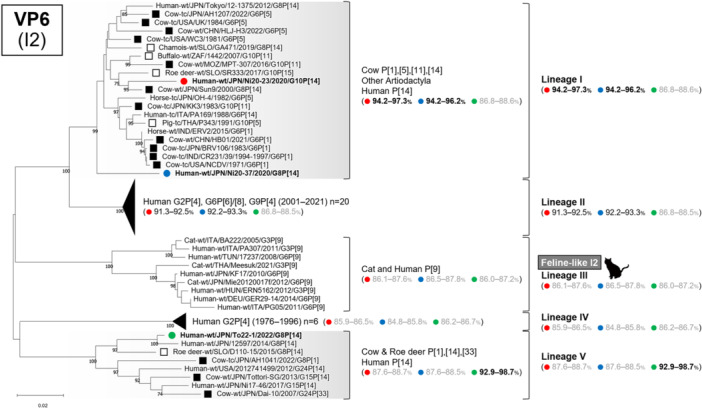
Phylogenetic trees of the VP6 (I2) gene of the study strains and representative RVA strains. The phylogenetic trees were constructed with the maximum likelihood method and 1000 bootstrap replicates, using the MEGA 11 software package. Bootstrap values of ≥ 70% are indicated at each node. The genetic distance (nucleotide substitutions per site) is indicated at the bottom of the trees. Strains Ni20‐23, Ni20‐37, and To22‐1 are represented by red, blue, and green filled circles, respectively. Bovine strains and other artiodactyl strains are represented by black‐filled squares and black squares, respectively. Nucleotide identities between strains Ni20‐23, Ni20‐37, and To22‐1 and other strains are shown in parentheses.

**FIGURE 5:**
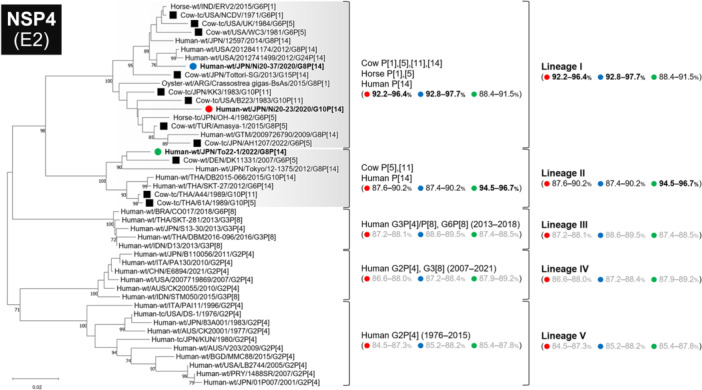
Phylogenetic trees of the NSP4 (E2) gene of the study strains and representative RVA strains. The phylogenetic trees were constructed with the maximum likelihood method and 1000 bootstrap replicates, using the MEGA 11 software package. Bootstrap values of ≥ 70% are indicated at each node. The genetic distance (nucleotide substitutions per site) is indicated at the bottom of the trees. Strains Ni20‐23, Ni20‐37, and To22‐1 are represented by red, blue, and green filled circles, respectively. Bovine strains and other artiodactyl strains are represented by black‐filled squares and black squares, respectively. Nucleotide identities between strains Ni20‐23, Ni20‐37, and To22‐1 and other strains are shown in parentheses.

Phylogenetic analyses of all 11 genome segments confirmed that all three strains belonged to artiodactyl lineages (Figures 2, 3, 4, 5, Supplementary Figure 1A–G).

### 3.6 VP7 Tree (G10 and G8)

The VP7 tree classified G10 strains into three lineages, all including artiodactyl strains (Figure 2A). Ni20‐23 clustered in lineage I with Japanese bovine P [11] strains from 2019 to 2020 (e.g., HKD88, OKY47, FKS94), showing 97.9%–99.3% identity [26]. By contrast, nucleotide identities with lineage II, which includes Argentine bovine P [5] and P [11] strains, and lineage III, including bovine strains from India, China, and Turkey, were much lower, showing 84.3%–85.7% identity.

G8 strains formed four lineages (Figure 2B), and strains Ni20‐37 and To22‐1 belonged to lineage I. Ni20‐37 clustered with Japanese and Korean bovine strains (e.g., Niigata9801, KJ56‐1, KJ11, 2011HGS) and Korean porcine strains (e.g., 174‐1, 06‐46‐2), sharing 97.3%–98.8% identity [27]. To22‐1 clustered with bovine strain JPN/Tokushima9503/1995 [27], alpaca strain PER/562/2010, and human strains AUS/WAG8.1/2002 [28], JPN/12597/2014 [25], and USA/2012841174/2012 [29], showing 96.7%–98.4% identity. By contrast, nucleotide identities between Ni20‐37 and To22‐1 and the other three lineages (lineage II, including Turkish bovine P [5] strains; lineage III, including African human P [4] and P [6] strains; lineage IV, including human DS‐1‐like P [8] strains from 2013 to 2021 and Thai bovine P [1] strains from the 1980s) were all below 85%.

Overall, VP7 phylogenetic analysis indicated that all three strains clustered within artiodactyl‐associated G10 or G8 lineages and were clearly separated from typical human RVA lineages.

### 3.7 VP4 Tree (P[14])

The VP4 tree divided P [14] strains into two distinct lineages: lineage I (“Artiodactyl‐like P[14]”) comprising artiodactyl and human strains as well as one fox strain, and lineage II (“Lapine‐like P[14]”), comprising rabbit and human strains (Figure 3). All three study strains (Ni20‐23, Ni20‐37, To22‐1) belonged to lineage I. Ni20‐37 and To22‐1 clustered together, while Ni20‐23 belonged to a distinct sub‐lineage within lineage I. The nucleotide identities between the three strains in this study and typical human strains Wa (P[8]) and DS‐1 (P[4]), as well as artiodactyl P [1], P [5], P [7], and P [11] strains, were all below 63%, indicating distant genetic relationships.

Thus, all three strains belonged to the artiodactyl‐like P [14] lineage and were genetically distinct from both lapine‐like P [14] and common human RVA lineages.

### 3.8 VP6 Tree (I2)

The VP6 tree comprised five lineages, with artiodactyl strains split between lineages I and V (Figure 4). Strains Ni20‐23 and Ni20‐37 belonged to lineage I with artiodactyl strains isolated worldwide from the 1970s–2020s, whereas To22‐1 clustered in lineage V with Japanese bovine and human P [14] strains isolated from the 2000s–2020s. Human strains from the 2000s belonged to lineage II, feline P [9] strains formed lineage III (“Feline‐like I2”), and older human G2P [4] DS‐1 strains were in lineage IV.

These results indicate that all three strains possessed artiodactyl‐associated VP6 segments, although they were distributed across two distinct artiodactyl lineages.

### 3.9 NSP4 Tree (E2)

The NSP4 tree formed five lineages, with artiodactyl strains distributed in lineages I and II (Figure 5). Strains Ni20‐23 and Ni20‐37 belonged to lineage I with artiodactyl strains from the United States, Japan, India, and Turkey, whereas To22‐1 belonged to lineage II with bovine strains from Denmark and Thailand. All three strains shared < 90% identity with human RVA lineages III–V.

Accordingly, NSP4 genes of all three strains clustered within artiodactyl‐associated lineages and were distinct from typical human RVA NSP4 lineages.

### 3.10 Seven Other Genetic Trees

In the VP1 (R2) tree, four lineages were identified, with artiodactyl strains distributed in lineages I and II (Supplementary Figure 1A). All three strains fell within lineage I.

In the VP2 (C2) tree, five lineages were present, with artiodactyl strains found in lineages I–III (Supplementary Figure 1B). Strains Ni20‐23 and Ni20‐37 grouped in lineage I, while To22‐1 clustered with Argentine bovine strain B383 in lineage III.

In the VP3 (M2) tree, four lineages were observed, with all artiodactyl strains assigned to lineage I (Supplementary Figure 1C). All three strains belonged to this lineage.

In the NSP1 (A3) tree, two major lineages were evident: lineage I, including worldwide artiodactyl strains (“Artiodactyl‐like A3”), and lineage II, comprising feline and human P [9] strains (“Feline‐like A3”) (Supplementary Figure 1D). The three strains clustered within lineage I.

In the NSP2 (N2) tree, seven lineages have been described, with artiodactyl strains distributed mainly in lineages II, III, and VII (Supplementary Figure 1E). Strain Ni20‐23 belonged to lineage II with bovine strains from the 1970–2010s. Strains Ni20‐37 and To22‐1 grouped with Japanese and Korean bovine strains in lineage VII.

In the NSP3 (T6) tree, most strains originated from artiodactyl hosts and clustered in lineages I and II (Supplementary Figure 1F). The three strains were in lineage I.

In the NSP5 (H3) tree, four lineages were observed, with artiodactyl strains belonging to lineages I, II, and IV (Supplementary Figure 1G). Strain Ni20‐23 was in lineage II, and strains Ni20‐37 and To22‐1 were in lineage IV.

Collectively, phylogenetic analyses of the remaining seven genome segments consistently placed all three strains within artiodactyl‐associated lineages, with lineage‐level variation among individual genes.

### 3.11 Comparison of the Amino Acid Sequences of the VP7 and VP4 Protein

Strain To22‐1/G8P [14] was detected in a child who had received two doses of Rotarix. The nucleotide identity of the VP7 and VP4 genes between To22‐1 and the Rotarix (G1P[8]) vaccine strain was only 69.1% and 58.5%, indicating substantial genetic divergence. Therefore, we compared the VP7 and VP4 amino acid sequences of the three RVA strains identified in this study with those of the Rotarix and RotaTeq vaccine strains, focusing on known antigenic epitopes.

### 3.12 VP7 Protein

The VP7 protein contains three antigenic epitopes (7‐1a, 7‐1b, and 7‐2), comprising a total of 29 amino acid residues [30]. Among these, 22 residues are known neutralization escape sites [30].

**FIGURE 6:**
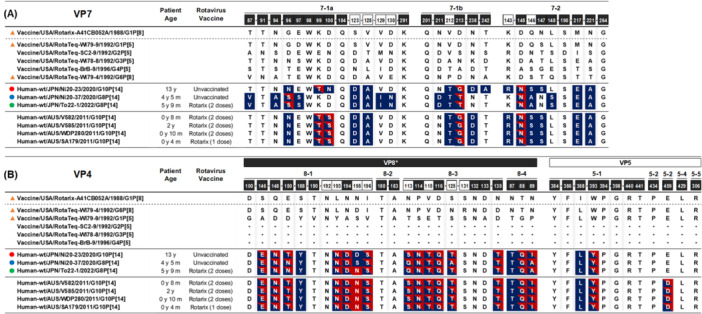
Amino acid substitutions within antigenic epitopes encoded by the VP7 and VP4 genes. Panel (A) shows VP7, and panel (B) shows VP4. Amino acid residues shown in white text within black squares represent residues previously demonstrated to mediate neutralization escape. Amino acids highlighted in indigo and red differ from those in the Rotarix® and RotaTeq® vaccine strains, respectively. Among the five RotaTeq® strains, four strains containing P [5] share identical amino acids at all 37 residues in VP4.

Compared with the G1P [8] Rotarix vaccine strain, Ni20‐23 showed 15 amino acid substitutions (51.7%, 15/29), including 10 at known neutralization escape site (45.4%, 10/22). Ni20‐37 and To22‐1 each showed 16 amino acid substitutions (55.2%, 16/29), also including 10 at neutralization escape sites (45.4%, 10/22) (Figure 6A).

Compared with the RotaTeq vaccine strain, all three study strains exhibited 3 amino acid substitutions (10.3%, 3/29) at known neutralization escape sites.

For reference, several G10P [14] strains detected in Rotarix‐vaccinated patients with acute gastroenteritis in Australia in 2011 (V582, V585, WDP280, and SA179) also exhibited 14 amino acid substitutions relative to the Rotarix strain (48.3%, 14/29), 9 of which corresponded to known neutralization escape sites (40.9%, 9/22) [31].

### 3.13 VP4 Protein

The VP4 protein contains nine antigenic epitopes: four (8–1 to 8–4) located in the VP8* region and five (5–1 to 5–5) located in the VP5* region, comprising a total of 37 amino acid residues [28]. Among these, 29 residues are known neutralization escape sites [30].

Compared with the G1P [8] Rotarix vaccine strain, Ni20‐23 and Ni20‐37 each exhibited 19 amino acid substitutions (51.3%, 19/37), including 13 at known neutralization escape sites (44.8%, 13/29). To22‐1 showed 18 amino acid substitutions (48.6%, 18/37), also including 13 at neutralization escape sites (44.8%, 13/29) (Figure 6B).

Compared with the RotaTeq vaccine strain, Ni20‐23 and Ni20‐37 each exhibited 15 amino acid substitutions (40.5%, 15/37), including 10 at known neutralization escape sites (34.4%, 10/29). To22‐1 showed 16 amino acid substitutions (43.2%, 16/37), also including 10 at neutralization escape sites (34.4%, 10/29).

Previously reported Australian G10P [14] strains (V582, V585, WDP280, and SA179) also exhibited 19 amino acid substitutions relative to the Rotarix strain (51.3%, 19/37), 14 of which corresponded to known neutralization escape sites (48.2%, 14/29) [31].

## 4 Discussion

In this study, we performed whole‐genome analyses of three rare RVA strains: one G10P [14] and two G8P [14], which were detected in pediatric gastroenteritis patients in Hokkaido Prefecture, Japan, during 2020 and 2022. All three strains shared a consistent genotype constellation (G10/G8‐P[14]‐I2‐R2‐C2‐M2‐A3‐N2‐T6‐E2‐H3), and phylogenetic analyses across all 11 genome segments confirmed that they belonged to artiodactyl‐like lineages. Although the strains displayed identical genotype constellations, none exhibited high nucleotide identity across all 11 genome segments, indicating that they may not share a recent common ancestor (Figure 1). However, because currently available artiodactyl‐like RVA sequences remain limited, the absence of closely related reference strains may reflect gaps in animal RVA surveillance, rather than the true absence of precursor viruses.

Human infections with artiodactyl‐like RVAs have been reported previously, most of which involve the P [14] VP4 genotype [13, 20, 21, 22, 23, 25, 28, 29, 32, 33, 34]. By contrast, other VP4 genotypes commonly found in artiodactyls, such as P [1], P [5], P [7], or P [11], are rarely detected in humans, suggesting that P [14] may facilitate cross‐species transmission. In addition to artiodactyl species, a few reports have also documented the detection of P [14] RVAs in rabbits [35, 36, 37]. Phylogenetic analysis of the VP4 P [14] gene revealed that artiodactyl and rabbit strains form distinct lineages; however, human P [14] strains were found in both lineages (Figure 3). Moreover, although only one case has been reported, a G8P [14] strain (L157‐VS) detected in a red fox in Croatia in 2018 [38] clustered within lineage I, together with artiodactyl‐like P [14] strains. These findings suggest that multiple animal species, including artiodactyls, may serve as reservoirs for human P [14] infections.

Conversely, the broad diversity of VP7 genotypes among artiodactyl‐like RVA strains detected in humans (including G3, G4, G6, G8, G10, G15, and G24) indicates that VP7 alone may not fully determine zoonotic transmission potential. However, phylogenetic analysis of the VP7 (G8) gene showed that human G8P [14] strains (lineage I) form a distinct cluster, separate from G8 strains associated with human P genotypes (P [4], P [6], and P[8]; lineages II–IV) (Figure 2B). This finding suggests that VP7 may still contribute to host range in combination with other genomic factors, and that only certain VP7 lineages may be compatible with P [14]. Taken together, these observations imply that zoonotic spillovers are likely shaped by multiple interacting determinants rather than a single gene segment.

In addition to P [14], the VP4 genotype P [9], derived from feline RVAs, is another example of zoonotic transmission to humans. Close contact with cats and the presence of histo‐blood group antigen type A have been proposed as risk factors for P [9] infection [39, 40]. We previously reported a 3‐year‐old girl living with multiple cats who developed gastroenteritis caused by a feline‐like G3P [9] RVA strain [41]. These observations highlight the importance of integrating clinical and molecular findings to better understand host‐range restrictions. By contrast, factors enabling P [14] infection in humans remain unclear. In this study, the three patients had no documented animal contact and resided in urban areas (Sapporo city and Tomakomai city) with limited artiodactyl populations, suggesting that alternative intermediate hosts or indirect environmental transmission routes should not be ruled out. This emphasizes the importance of continued surveillance across human, animal, and environmental sources. In addition, alternative mechanisms such as VP4 receptor‐binding differences, host susceptibility factors, and early adaptation of animal strains in the human intestine may also contribute to the spillover of P [14] RVAs, and these possibilities merit further investigation.

Regarding vaccination, Ni20‐23 and Ni20‐37 were isolated from unvaccinated children, whereas To22‐1 (G8P[14]) was detected in a child who had received two doses of Rotarix. Similar cases were reported in Australia in 2011, where four Rotarix‐vaccinated children aged 0–2 years developed gastroenteritis caused by artiodactyl‐like G10P [14] RVA strains [31]. As shown in Figure 6, approximately half of the amino acid residues within the antigenic regions of VP7 and VP4 differed between the Rotarix vaccine strain (a live‐attenuated human G1P [8] virus) and the P [14] strains examined in this study. This antigenic divergence suggests a potential antigenic mismatch with current vaccines; however, functional neutralization assays are needed to verify whether this affects protection.

Although human infections with artiodactyl‐like RVA are generally sporadic, a 2012 school trip outbreak in Japan involved a G8P [14] strain (Tokyo/12‐1375) with the same genotype constellation as Ni20‐37 and To22‐1 [22]. Although animal contact histories were not documented, this event suggests the potential for human‐to‐human transmission of artiodactyl‐like G8P [14] strains. No further outbreaks with similar strains have been reported in Japan or globally, but continued surveillance is warranted.

## 5 Conclusion

This study reports three pediatric gastroenteritis cases in Japan caused by artiodactyl‐like P [14] RVA strains, including one Rotarix‐vaccinated child. Our findings highlight the genetic diversity and zoonotic potential of P [14] RVA strains and raise the possibility of antigenic mismatch with current vaccines, underscoring the need for continued evaluation of vaccine cross‐reactivity. Continuous surveillance and further studies will be essential to elucidate the mechanism underlying interspecies transmission of animal‐derived P [14] RVAs to humans, to assess vaccine effectiveness, and to evaluate the potential risk of human outbreaks.

## Author Contributions

Akira Takebayashi and Yuya Fukuda were involved in conceptualization, methodology, data curation, formal analysis, and writing the original draft. These two authors contributed equally to this work. Yoshiki Fujii was involved in methodology, data curation, formal analysis, funding acquisition, and investigation. Hiroshi Nihira, Minako Kihara, Yoshimasa Kudou, Shuhei Adachi, and Noritaka Shintani were involved in the investigation. Takeshi Tsugawa was involved in conceptualization, methodology, project administration, and supervision. All authors revised the manuscript, approved the manuscript for publication, and agreed to be accountable for all aspects of the work and to ensure that issues related to the accuracy or integrity of any part of the work are appropriately investigated and resolved.

## Conflicts of Interest

The authors declare no conflicts of interest.

## Supporting information


**Supplement Figure 1:** Phylogenetic trees of the VP1 (R2), VP2 (C2), VP3 (M2), NSP1 (A3), NSP2 (N2), NSP3 (T6), and NSP5 (H3) genes of the study strains and representative RVA strains. The phylogenetic trees were constructed with the maximum likelihood method and 1000 bootstrap replicates, using the MEGA 11 software package. Bootstrap values of ≥ 70% are indicated at each node. The genetic distance (nucleotide substitutions per site) is indicated at the bottom of the trees. Strains Ni20‐23, Ni20‐37, and To22‐1 are represented by red, blue, and green filled circles, respectively. Bovine strains and other artiodactyl strains are represented by black filled squares and black squares, respectively. Nucleotide identities between strains Ni20‐23, Ni20‐37, and To22‐1 and other strains are shown in parentheses.

## Data Availability

The data that support the findings of this study are available from the corresponding author, Takeshi Tsugawa, upon reasonable request.
