# Supplementary figures and images for: Whole‐Genome Characterization of Rare Artiodactyl‐Like G10P [14] and G8P [14] Rotavirus a Strains Detected in Pediatric Gastroenteritis Cases in Hokkaido Prefecture, Japan
Source: J Med Virol. 2026 Mar 18;98(3):e70875. doi: 10.1002/jmv.70875 (PMC12997524; doi:10.1002/jmv.70875)

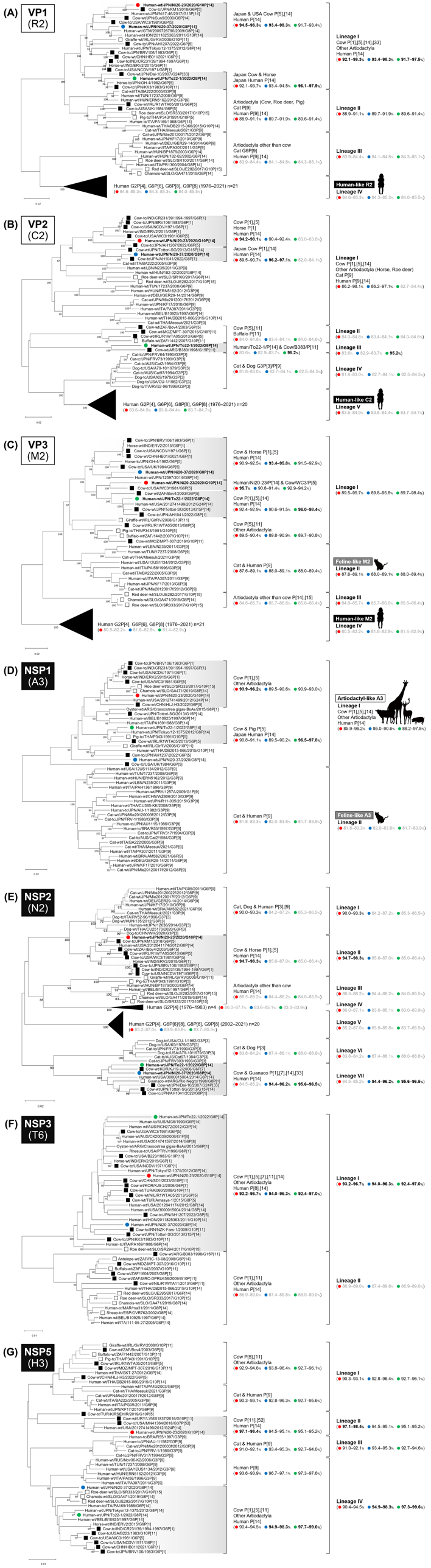

Supplement: Supplementary file 1 — Supplement Figure 1: Phylogenetic trees of the VP1 (R2), VP2 (C2), VP3 (M2), NSP1 (A3), NSP2 (N2), NSP3 (T6), and NSP5 (H3) genes of the study strains and representative RVA strains. The phylogenetic trees were constructed with the maximum likelihood method and 1000 bootstrap replicates, using the MEGA 11 software package. Bootstrap values of ≥ 70% are indicated at each node. The genetic distance (nucleotide substitutions per site) is indicated at the bottom of the trees. Strains Ni20‐23, Ni20‐37, and To22‐1 are represented by red, blue, and green filled circles, respectively. Bovine strains and other artiodactyl strains are represented by black filled squares and black squares, respectively. Nucleotide identities between strains Ni20‐23, Ni20‐37, and To22‐1 and other strains are shown in parentheses. [file JMV-98-e70875-s001.png]
